# Supplementary material for: Long-term trends in the incidence of peritoneal dialysis-related peritonitis disclose an increasing relevance of streptococcal infections: A longitudinal study
Source: PLoS One. 2020 Dec 21;15(12):e0244283. doi: 10.1371/journal.pone.0244283 (PMC7751873; doi:10.1371/journal.pone.0244283)
Supplement: Attachment — Submitted filename: PONE-D-20-22429_reviewer with highlights.pdf [file pone.0244283.s001.pdf]

PLOS ONE

The Growing Role of Streptococci as Causative Agents of Peritoneal Dialysis-related Peritonitis. A Longitudinal Analysis

--Manuscript Draft--

**Manuscript Number:**	PONE-D-20-22429
**Article Type:**	Research Article
**Full Title:**	The Growing Role of Streptococci as Causative Agents of Peritoneal Dialysis-related Peritonitis. A Longitudinal Analysis
**Short Title:**	Streptococcal peritoneal infections in PD
**Corresponding Author:**	Miguel Pérez Fontán, PhD<br>Complexo Hospitalario Universitario A Coruna<br>A Coruña, SPAIN
**Keywords:**	Peritoneal Dialysis; Peritoneal infection; Streptococcus; Incidence; Outcome
**Abstract:**	Background: The selective impact of strategies for prevention of PD-related peritonitis (PDrP) may have modified, in the long term, the causal spectrum, clinical presentation and outcomes of these infections.<br>Objectives: To compare trends in the incidence of PDrP by different microorganisms during a 30-year period, with a particular focus on streptococcal infections. To analyze the clinical presentation and outcomes of these infections. Secondarily, to investigate how the isolation of different species of streptococci can influence the clinical course of PDrP by this genus of bacteria.<br>Method: Following a retrospective, observational design we investigated 1061 PDrP (1990-2019). We used joinpoint regression analysis to explore trends in the incidence of PDrP by different microorganisms, and compared the risk profile (Cox), clinical presentation and outcomes (logistic regression) of these infections.<br>Main results: We disclosed significant trends to a decrease in the incidences of PDrP by staphylococci and Gram negative bacteria, while the absolute rates of streptococcal (average annual percent change +1,6%, 95% CI -0,1/+3,2) and polymicrobial (+1,8%, +0,1/+3,5) infections tended to increase, during the same period. Remarkably, streptococci were isolated in 58,6% of polymicrobial infections, and patients who suffered a streptococcal PDrP had a 35,8% chance of presenting at least one other infection by the same genus. The risk profile for streptococcal infections was comparable to that observed for PDrP overall. Streptococcal PDrP associated a severe initial inflammatory response, but their clinical course was generally nonaggressive thereafter. We did not observe a differential effect of different groups of streptococci on the clinical presentation or outcome of PDrP.<br>Conclusions: The last three decades have contemplated a growing involvement of streptococci as causative agents of PDrP. This behaviour suggests that current measures of prevention of PDrP may not be sufficiently effective, in the case of this genus of microorganisms.
**Order of Authors:**	Joana Eusebio Santos
	Catuxa Rodriguez-Magarinos
	Leticia Garcia Gago
	Daniela Astudillo Jarrin
	Sonia Pértega Diaz
	Ana Rodriguez-Carmona
	Teresa Garcia Falcon
	Miguel Pérez Fontán, PhD
**Additional Information:**	
**Question**	**Response**
**Financial Disclosure**	The authors received no specific funding for this work

Powered by Editorial Manager® and ProduXion Manager® from Aries Systems Corporation

Enter a financial disclosure statement that describes the sources of funding for the work included in this submission. Review the submission guidelines for detailed requirements. View published research articles from *PLOS ONE* for specific examples.<br><br>This statement is required for submission and **will appear in the published article** if the submission is accepted. Please make sure it is accurate.<br><br>**Unfunded studies**<br>Enter: *The author(s) received no specific funding for this work.*<br><br>**Funded studies**<br>Enter a statement with the following details:<br>• Initials of the authors who received each award<br>• Grant numbers awarded to each author<br>• The full name of each funder<br>• URL of each funder website<br>• Did the sponsors or funders play any role in the study design, data collection and analysis, decision to publish, or preparation of the manuscript?<br>• **NO** - Include this sentence at the end of your statement: *The funders had no role in study design, data collection and analysis, decision to publish, or preparation of the manuscript.*<br>• **YES** - Specify the role(s) played.<br><br>* typeset	
**Competing Interests**<br><br>Use the instructions below to enter a competing interest statement for this submission. On behalf of all authors, disclose any competing interests that could be perceived to bias this work—acknowledging all financial support and any other relevant financial or non-financial competing interests.<br><br>This statement **will appear in the published article** if the submission is accepted. Please make sure it is accurate. View published research articles	The authors have declared that no competing interests exist

from *PLOS ONE* for specific examples.<br><br>**NO authors have competing interests**<br><br>Enter: *The authors have declared that no competing interests exist.*<br><br>**Authors with competing interests**<br><br>Enter competing interest details beginning with this statement:<br><br>*I have read the journal's policy and the authors of this manuscript have the following competing interests: [insert competing interests here]*<br><br>* typeset	
**Ethics Statement**<br><br>Enter an ethics statement for this submission. This statement is required if the study involved:<br><br>• Human participants<br>• Human specimens or tissue<br>• Vertebrate animals or cephalopods<br>• Vertebrate embryos or tissues<br>• Field research<br><br>Write "N/A" if the submission does not require an ethics statement.<br><br>General guidance is provided below. Consult the submission guidelines for detailed instructions. **Make sure that all information entered here is included in the Methods section of the manuscript.**	This study was evaluated and approved by the joint Ethical Committee of A Coruña and Ferrol University Hospitals (code 2020/190).<br>Oral informed consent was requested and obtained from all the participants active at the time of this retrospective analysis . Data bases were fully anonymized for data processing.

Powered by Editorial Manager® and ProduXion Manager® from Aries Systems Corporation

**Format for specific study types**<br><br>**Human Subject Research (involving human participants and/or tissue)**<br>• Give the name of the institutional review board or ethics committee that approved the study<br>• Include the approval number and/or a statement indicating approval of this research<br>• Indicate the form of consent obtained (written/oral) or the reason that consent was not obtained (e.g. the data were analyzed anonymously)<br><br>**Animal Research (involving vertebrate animals, embryos or tissues)**<br>• Provide the name of the Institutional Animal Care and Use Committee (IACUC) or other relevant ethics board that reviewed the study protocol, and indicate whether they approved this research or granted a formal waiver of ethical approval<br>• Include an approval number if one was obtained<br>• If the study involved *non-human primates*, add *additional details* about animal welfare and steps taken to ameliorate suffering<br>• If anesthesia, euthanasia, or any kind of animal sacrifice is part of the study, include briefly which substances and/or methods were applied<br><br>**Field Research**<br><br>Include the following details if this study involves the collection of plant, animal, or other materials from a natural setting:<br>• Field permit number<br>• Name of the institution or relevant body that granted permission	
**Data Availability**<br><br>Authors are required to make all data underlying the findings described fully available, without restriction, and from the time of publication. PLOS allows rare exceptions to address legal and ethical concerns. See the PLOS Data Policy and FAQ for detailed information.	Yes - all data are fully available without restriction

A Data Availability Statement describing where the data can be found is required at submission. Your answers to this question constitute the Data Availability Statement and **will be published in the article**, if accepted.<br><br>**Important:** Stating 'data available on request from the author' is not sufficient. If your data are only available upon request, select 'No' for the first question and explain your exceptional situation in the text box.<br><br>Do the authors confirm that all data underlying the findings described in their manuscript are fully available without restriction?	
**Describe where the data may be found in full sentences. If you are copying our sample text, replace any instances of XXX with the appropriate details.**<br><br>• If the data are **held or will be held in a public repository**, include URLs, accession numbers or DOIs. If this information will only be available after acceptance, indicate this by ticking the box below. For example: *All XXX files are available from the XXX database (accession number(s) XXX, XXX.*).<br>• If the data are all contained **within the manuscript and/or Supporting Information files**, enter the following: *All relevant data are within the manuscript and its Supporting Information files.*<br>• If neither of these applies but you are able to provide **details of access elsewhere**, with or without limitations, please do so. For example:<br><br>*Data cannot be shared publicly because of [XXX]. Data are available from the XXX Institutional Data Access / Ethics Committee (contact via XXX) for researchers who meet the criteria for access to confidential data.*<br><br>*The data underlying the results presented in the study are available from (include the name of the third party*	Data cannot be shared publicly due to legal restrictions related to data protection. Requests should be addressed across the corresponding Ethical Committee (current secretary Natalia Cal Purriños)(ceic@sergas.es)

and contact information or URL).<br>• This text is appropriate if the data are owned by a third party and authors do not have permission to share the data.<br><br>* typeset	
Additional data availability information:	Tick here if the URLs/accession numbers/DOIs will be available only after acceptance of the manuscript for publication so that we can ensure their inclusion before publication.

Powered by Editorial Manager® and ProduXion Manager® from Aries Systems Corporation

Manuscript

Click here to access/download;Manuscript;aaaaPD_Strept_POne.docx

The Growing Role of Streptococci as Causative Agents of Peritoneal Dialysis-related Peritonitis. A Longitudinal Analysis

Joana Eugénio Santos[1], Catuxa Rodríguez Magariños[2], Leticia García Gago[2], Daniela Astudillo Jarrín[2], Sonia Pértega[3], Ana Rodríguez-Carmona[2], Teresa García Falcón[2], Miguel Pérez Fontán[2,4]

[1] Centro Hospitalario Espirito Santo, Evora (Portugal);

[2]Division of Nephrology, University Hospital A Coruña, Spain

[3]Division of Epidemiology, University Hospital A Coruña, Spain

[4]Health Sciences Faculty, University of A Coruña, Spain

CORRESPONDENCE: Miguel Pérez Fontán

Division of Nephrology

University Hospital A Coruña

Xubias 84

15006 A Coruña (Spain)

Phone & fax 34-981178159

Mail: miguel.perez.fontan@sergas.es

Short title: Streptococcal peritoneal infections in PD

Key words: Peritoneal Dialysis; Peritoneal infection; Streptococcus; Incidence; Outcome

Abstract

Background: The selective impact of strategies for prevention of PD-related peritonitis (PDrP) may have modified, in the long term, the causal spectrum, clinical presentation and outcomes of these infections.

Objectives: To compare trends in the incidence of PDrP by different microorganisms during a 30-year period, with a particular focus on streptococcal infections. To analyze the clinical presentation and outcomes of these infections. Secondarily, to investigate how the isolation of different species of streptococci can influence the clinical course of PDrP by this genus of bacteria.

Method: Following a retrospective, observational design we investigated 1061 PDrP (1990-2019). We used joinpoint regression analysis to explore trends in the incidence of PDrP by different microorganisms, and compared the risk profile (Cox), clinical presentation and outcomes (logistic regression) of these infections.

Main results: We disclosed significant trends to a decrease in the incidences of PDrP by staphylococci and Gram negative bacteria, while the absolute rates of streptococcal (average annual percent change +1,6%, 95% CI -0,1/+3,2) and polymicrobial (+1,8%, +0,1/+3,5) infections tended to increase, during the same period. Remarkably, streptococci were isolated in 58,6% of polymicrobial infections, and patients who suffered a streptococcal PDrP had a 35,8% chance of presenting at least one other infection by the same genus. The risk profile for streptococcal infections was comparable to that observed for PDrP overall. Streptococcal PDrP associated a severe initial inflammatory response, but their clinical course was generally nonaggressive thereafter. We did not observe a differential effect of different groups of streptococci on the clinical presentation or outcome of PDrP.

Conclusions: The last three decades have contemplated a growing involvement of streptococci as causative agents of PDrP. This behaviour suggests that current measures of prevention of PDrP may not be sufficiently effective, in the case of this genus of microorganisms.

Introduction

Peritonitis (PDrP) represents one of the most feared complications of chronic Peritoneal Dialysis (PD), associating significant rates of mortality [1,2] and PD technique failure [3-6]. The incidence of PDrP declined markedly between 1985 and 1995, after the introduction of Y-set, double bag systems, but improvements have been slower thereafter [5,7], and rates as high as one episode every two patient-years are still considered acceptable by current standards [8]. Singular advances, including the introduction of low glucose degradation products-based (low-GDP) solutions, may have been beneficial, but their impact has been disappointing, in clinical terms [9]. On the contrary, comprehensive approaches to prevention, including continuous quality improvement strategies, have provided a renewed hope of progress [10], and may mark the route to an optimized control of these infections.

Staphylococci are still viewed as the main source of PD-related PDrP [1,11,12]. However, a selective impact of preventive measures, preferentially oriented to reduce the consequences of touch contamination and catheter-related infections, may have influenced the etiologic spectrum and the strategies of management of PDrP [8], because these measures do not protect evenly from infections by different microorganisms. In particular, streptococcal infections have been a subject of limited attention in the past, due to the perceptions that these infections are relatively infrequent (with reported relative incidences of 5-12% of all PDrP) and follow a relatively benign clinical course [13-16]. On the other hand, the taxonomy and nomenclature of the ubiquitous genus *Streptococcus* has been reviewed in the last years [17] and, aside from single case reports, information on the compared aggressiveness of PDrP by different members of this family of microorganisms is scarce [18]. Finally, streptococci may be a common component of polymicrobial PDrP, but the real incidence of this circumstance and its clinical significance are also unclear.

Following an observational, retrospective design, we have investigated time trends in the incidence of PDrP in our centre during a period of 30 years. Our main interest was to

pursue a clinical perception that advances in the prevention and management of PDrP could have modified the clinical scenery of PD-related peritonitis, conferring streptococci a progressively dominant role, with potential consequences for the clinical presentation, management and outcomes of these infections.

Method

General design

Following an observational, retrospective design, we analyzed trends in the incidence and outcomes of PDrP diagnosed in our centre between January 1990 and December 2019. Our main focus was to disclose changes in the incidences of PDrP by different causative agents, with a particular interest in streptococcal infections (primary objective). For this purpose, we categorized the study follow-up into six periods (1990-94, 1995-99, 2000-04, 2005-09, 2010-14 and 2015-19). We also analyzed the clinical presentation, treatment and outcomes of the aforementioned groups, with streptococcal infections again as the main reference (primary objectives too). The main clinical outcome was a composite of PDrP-related death, drop-out to hemodialysis for more than 3 months and need for catheter removal to control the infection (treatment failure). Secondarily, we investigated the clinical significance of PDrP by different species of streptococci, as also the role of these bacteria in polymicrobial infections (secondary objectives).

The study complied with the principles of the Declaration of Helsinki and the ethical requirements of our centre for retrospective observational studies. Informed consent was a requisite for inclusion of eligible patients who were accessible at the time of initiation of the study. The study protocol was evaluated and approved by the local ethical committee of A Coruña-Ferrol hospitals (code 2020/190).

Study population

We recruited for this study all patients starting PD in our unit between January 1, 1990 and October 1, 2019. Follow-up was closed by December 31, 2019. We excluded from analysis patients <18 years of age, those with a follow-up on PD <1 month, and those with inadequate clinical records. No patient denied consent for participation.

Study variables and strategy of analysis

Our interest was addressed to episodes of PDrP occurring during the study period. In particular, we focused on streptococcal infections, and used PDrP of other causes as control groups. For this purpose, we categorized the following study groups:

1) *Streptococcus spp*.
2) *Staphylococcus aureus* (SAu).
3) Coagulase-negative staphylococci (CNSt).
4) *Enterobacteriaceae* and nonfermenting Gram negative bacteria (GNB).
5) Polymicrobial PDrP, excluding those with a rampant intestinal origin (immediate surgical approach or isolation of multiple enteric microorganismos, including intestinal anaerobics)
6) Culture-negative infections.

We did not consider PDrP with primary isolation of yeasts (n=30), filamentous fungi (n=2) or mycobacteria (n=5), due to their low frequency and particular significance. PDrP caused by other less frequent (less than 50 cases) Gram positive [e.g. *Enterococcus spp* (n=35) or *Corynebacterium spp* (n=13)] or Gram negative bacteria [e.g. *Neisseria spp* (n=10)] were also excluded from analysis.

The main study variable was the causative organism of each PDrP, always with a main focus on streptococcal infections. We explored:

1) The time course of the incidence of PDrP by different causative microorganisms.

2) The risk profile for different types of PDrP, according to the demographic and clinical characteristics of the study population: age, gender, underlying kidney disease, diabetes mellitus, previous episodes of PDrP, treatment with immunosuppressants, PD vintage, PD modality, use of icodextrin, SAu carriage [19], comorbidity score (Charlson), malnutrition (subjective global assessment), socioeconomic status, assisted versus autonomous PD, body mass index (weight/height^2), hemoglobin, albumin (autoanalyzer), C-reactive protein (Immunoturbidimetry), glomerular filtration rate (GFR)(mean of urea and creatinine renal clearances), and peritoneal transport (D/P ratio of creatinine at 240´ D/Pcrea).
3) Clinical presentation of the PDrP: simultaneous catheter-related infection (catheter-related PDrP), hospitalization, peritoneal cell-count at baseline, and number of days until complete clinical, bacteriologic and cytologic remission or catheter removal.
4) Initial and susceptibility-oriented antimicrobial therapy
5) Outcomes of PDrP:
   - Peritoneal catheter removed
   - Transfer to hemodialysis for at least 3 months after the infection
   - Death for any reason during hospital admission or within 30 days after initiation of the episode.
   - Treatment failure, defined by any of the three previous outcomes (main clinical outcome).

PDrP was defined according to the ISPD guidelines [8,20]. The same sources were used for definition of relapsing, recurrent and repeat PDrP. In our Unit, hospital admission for PDrP is indicated for high-risk patients, infections with an aggressive clinical presentation, treatment failure after oriented antimicrobial therapy and isolation of microorganisms demanding in-centre management.

We used integrated Y systems for continuous ambulatory PD, and Home Choice (Baxter, Deerfield, IL, USA) cyclers for automated PD. Conventional, lactate-based PD solutions were used until March 2008, when patients were switched to low-GDP solutions. We have performed systematic screening of nasal (since 1990) and pericatheter (since 1996) carriage of SAu to patients and PD partners during the whole study period. Carriers were treated with nasal and pericatheter mupirocin. Our protocol for initial treatment of PDrP was based on intraperitoneal ciprofloxacin between 1990 and 2007. In September 2007, we switched to intravenous vancomycin + intraperitoneal cefotaxime, after susceptibility of CNSt to ciprofloxacin fell below acceptable standards.

Statistics

Continuous variables are expressed as mean ± SD, or as median (interquartile range), in case of markedly non-normal distribution. Categorical variables are presented as the number of cases (%). Basic univariate comparisons were produced by means of usual parametric [two-tailed Student's t test, ANOVA (Scheffé)] and nonparametric tests (χ^2 distribution, Mann Whitney, Spearman's correlation coefficient), as needed.

We used joinpoint regression analysis to disclose time trends in the overall and by-causative agent rates of incidence of PDrP, calculating the annual percent change (APC), and the average annual percent change (AAPC) for the whole study period [21]. The risk profile for PDrP by different families of microorganisms (survival to the first episode) was first explored by means of Kaplan Meier plots (log rank test), and then by stepwise Cox models. For Kaplan Meier analyses, we categorized numerical variables by tertiles. For multivariate analyses, we considered only first order interaction terms. PD vintage (categorized in three periods: 1990-99, 2000-09 and 2010-19) was systematically considered as a control variable in these multivariate models.

The clinical presentation and antimicrobial therapy characteristics are presented using a descriptive approach, including univariate comparisons. The same applies for outcomes, in which case we also carried out a multivariate approach (stepwise logistic regression), to categorize better the role of different bacteria, on these outcomes. Risk estimations are presented as Odds ratios (OR) (logistic regression) or hazard ratios (HRs)(Cox), with 95% confidence intervals (CI). A two-tailed p-value <0.05 was considered significant (the software used for time trends analyses does not provide exact p values).

We used the SPSS 19.0 software for general data management and statistic analyses. Time trends analyses were produced with the help of the Joinpoint Regression Program 4.7.0.0. (Feb 2019; Statistical Research and Applications Branch, National Cancer Institute).

Results

Overview and time trends

The study population included 878 patients, after excluding 25 other individuals who did not meet the inclusion criteria. Their main baseline characteristics are presented in Table 1. Four hundred and sixty-two patients (52.6%) suffered at least one episode of PDrP, and 158 (18.0%) suffered at least one streptococcal PDrP. The latter was the etiologic group with the highest incidence of repeat infection (35,8%), including 25 patients (15,8%) with more than two monobacterial streptococcal PDrP during follow-up (range 3-7). Only CNSt approached this figure (29,1%, including 12,6% with more than two episodes), which was markedly lower in the cases of SAu (11,6%), GNB (21,2%), polymicrobial (11,1%) and culture-negative PDrP (16,0%)(p=0,003). Median delays to the first episode of PDrP were 8 (interquartile range 3 to 17) (overall) and 12 months (interquartile range 5 to 24)(streptococcal PDrP).

Table 1

Baseline characteristics of the study population

Age (years)	59,2 (15,6)
Gender (males/females)(%)	522/356 (59,5/40,5)
Kidney disease	
Glomerular	117 (13,3)
Interstitial	97 (11,0)
Vascular (including nephroangiosclerosis)	84 (9,6)
Cystic	76 (8,7)
Systemic diseases (including paraproteinemias)	46 (5,2)
Diabetic nephropathy	264 (30,0)
Other/Unknown	194 (22,1)
Diabetes (%)	310 (39,4)
Charlson's score	4,1 (2,0)
Recent/Ongoing immunosuppression (%)	85 (9,7)
Malnutrition (%)	83 (9,4)
Body mass index (Kg/m^2)	26,0 (4,9)
Low socioeconomic status (%)	281 (32,0)
Family-assisted PD (%)	347 (39,6)
PD vintage (start 1990-94; 1995-99; 2000-04; 2005-09; 2010-14; 205-19)(%)	134/192/178/135/123/116 (15,3/21,9/20,3/15,4/14,0/13,2)
Modality of PD (Automated PD/CAPD)(%)	274/604 (31,2/68,8)
Icodextrin (%)	184 (21,0)
D/P creatinine 240', baseline PET (n=664)	67,9 (12,1)
Staphylococcus aureus carrier (%)	383 (43,6)
Glomerular filtration rate (mL/minute)	6,2 (4,1)
Hemoglobin (g/dL)	10,5 (1,6)
Plasma albumin (g/L)	37,1 (5,6)
Serum C reactive protein (mg/dL)(n=717)	0,59 (0,28-1,45)
Follow-up (months)	28,9 (25,7)

Figures denote mean values ± standard deviation, median with interquartile range (numerical variables) or absolute numbers (%) (categorical variables)
Keys: PD: Peritoneal Dialysis; CAPD: Continuous Ambulatory Peritoneal Dialysis; PET: Peritoneal equilibration test

Overall, we recorded 1061 episodes of PDrP during follow-up, caused by streptococci (n=235, 22,1%), SAu (n=57, 5,4%), CNSt (n=285, 26.9%) or GNB (n=175, 16,5%). We also registered 133 polymicrobial (12,5%) and 126 (11,9%) culture-negative infections. Regarding streptococcal infections, 223 were caused by viridans group streptococci and only 12 by non-viridans species. Among viridans streptococci, the *mitis* (including *oralis, sanguis and gordonii*)(n=92) and the *salivarius* (including *vestibularis*)(n=85) groups predominated. On the other hand, at least one streptococcal strain was isolated in 78 polymicrobial infections (58,6%)(Table 2). Streptococci were less frequently identified in the latter group between 1990 and 1999 (38,9%), but kept consistently above 60% after 2000 (p=0,016).

Table 2

Microorganisms isolated in polymicrobial peritonitis

**Microorganism**	**n**
Coagulase-negative staphylococci	55
Staphylococcus aureus	7
Streptococcus spp	116
Enterococcus spp	23
Enterobacteriaceae and nonfermenting Gram negative bacteria	83
Other	8

Table 3 displays the absolute and relative incidences of PDrP during the different study periods. Joinpoint analyses disclosed a progressive decrease in the incidence of PDrP overall. This tendence was supported by a declining incidence of infections by staphylococci and GNB (Table 3, Figure 1), while an opposite trend was observed for polymicrobial and streptococcal infections (not significant in the latter case)(Figure 1). Separate APC analyses showed rather homogeneous time courses for these trends, except in the cases of PDrP overall [AAPC -5,4%

per year 1990-1999 (95% CI -9,0/-1,6, p<0,001) versus -0,7% per year 2000-2019 (95% CI -1,7/+0,2)] and infections by *Staphylococcus aureus* [AAPC -12,4% per year 1990-1999 (95% CI -22,0/-1,6), 17,5% per year2000-2008 (95% CI -1,9/+40,8) and -21,7% per year 2009-2019 (95% CI -31,3/-10,7)].

Table 3

Time course of the absolute and relative rates of incidence of PD-related peritonitis of different etiologies

	**90-94**	**95-99**	**00-04**	**05-09**	**10-14**	**15-19**
Follow-up (months)	3558	4748	5495	4823	4182	4442
Follow-up (patient-years)	296,5	395,7	457,9	401,9	348,5	370,2
**ABSOLUTE RATES** (episodes/patient/year)						
**All infections** (n)	190	188	200	183	154	146
Incidence	0,64	0,48	0,44	0,45	0,44	0,39
Streptococcus spp (n)	31	25	50	36	48	45
Incidence	0,104	0,063	0,109	0,089	0,138	0,122
Staphylococcus aureus (n)	13	10	7	21	4	2
Incidence	0,043	0,025	0,015	0,052	0,001	0,005
**Coagulase negative *Staphylococcus spp*** (n)	69	59	59	51	26	21
Incidence	0,233	0,149	0,129	0,127	0,074	0,057
**Gram negative bacteria*** (n)	29	35	32	32	26	21
Incidence	0,098	0,088	0,070	0,079	0,074	0,057
**Polymicrobial** (n)	11	25	24	17	26	30
Incidence	0,037	0,063	0,052	0,042	0,074	0,081
**Negative culture** (n)	14	23	24	19	22	24
Incidence	0,047	0,058	0,052	0,047	0,063	0,065
**RELATIVE RATES** (% of all episodes of PDrP)						
Streptococcus spp	16,3	13,3	25,2	19,7	31,2	31,0
Staphylococcus aureus	6,8	5,3	3,5	11,5	2,6	1,4
**Coagulase negative *Staphylococcus spp***	36,3	31,6	29,7	28,0	16,9	14,5
**Gram negative bacteria***	15,3	18,7	16,0	17,5	16,9	14,5
**Polymicrobial**	5,8	13,3	12,0	9,3	16,9	20,7
**Culture-negative**	7,4	12,3	12,0	10,4	14,3	16,6

* *Enterobacteriaceae* + Nonfermenting Gram negative bacteria

Risk profile for PDrP

Kaplan Meier analysis disclosed correlations between the probability of presenting PDrP (any cause) during follow-up, on one side, and older age (p=0,023 log rank), higher Charlson's score (p=0,008), lower GFR (p=0,035), higher C-reactive protein levels (p=0,012), higher body mass index (p=0,035), and family-assisted PD (p=0,045), on the other. Patients on automated PD also exhibited a trend to a lower risk of infection than those on CAPD (p=0,07). On the other hand, the risk of suffering streptococcal PDrP was univariately linked to older age (p=0,003), lower plasma albumin (p=0,041), higher body mass index (p=0,021), lower blood haemoglobin (p=0,047) and automated PD rather than CAPD (p=0,050). PD vintage was a univariate predictor of the risk of streptococcal (p=0,004), but not overall PDrP (p=0,124)(other variables displayed in Table 1 not significant).

Table 4 shows the multivariate (Cox) risk profile for PDrP overall, and streptococcal PDrP. Both models were quite similar, except for a clearly stronger impact of PD vintage on the risk of streptococcal infection, and a more marked association of baseline GFR with the later risk of PDrP overall.

Table 4

Risk profile for peritoneal infection (overall) and streptococcal peritonitis. Multivariate analysis

	Overall			Streptococcal		
	**HR**	**95% CI**	**P value**	**HR**	**95% CI**	**P value**
PD vintage (ref. 1990-99)						
2000-09	1,21	0,94-1,57	0,11	1,71	1,22-2,62	0,009
2010-19	0,97	0,72-1,32	0,94	1,77	1,12-2,80	0,015
Age (per year)	1,01	1,00-1,03	0,026	1,02	1,01-1,04	0,001
Plasma albumin (per g/L)	0,98	0,96-0,99	0,021	0,96	0,93-0,99	0,011
GFR (per mL/min)	0,97	0,94-0,99	0,029	-	-	-
Body mass index (per Kg/m^2)	1,03	1,00-1,05	0,021	1,04	1,00-1,07	0,046

Stepwise Cox's regression. First-order interaction terms not significant. For the overall risk of PDrP, -2log likelihood 4429,30, $\chi 2$ 28,93, p<0,0005. For streptococcal PDrP, -2log likelihood 1692,86, $\chi 2$ 36,52, p<0,0005

Keys: HR: Hazard Ratio; CI: Confidence interval; PD: Peritoneal Dialysis; GFR: Glomerular Filtration Rate

Clinical presentation according to causative microorganism

The main clinical characteristics of PDrP by different causative microorganisms are displayed in Table 5. We did not record a single instance of streptococcal catheter-related PDrP between 1990 and 2019.

Table 5

Clinical presentation and antimicrobial therapy

	Strept.	SAu	CNS	GNB	Poly	Culture-negative	P value
Catheter-related (%)	0	**34,1**	**2,3**	**14,2**	0,8	0	0,0005
Hospitalization (%)	14,0	**40,4**	13,3	**33,0**	**26,7**	14,3	0,0005
Number of in-hospital days	10,7	16,3	13,3	**20,5**	12,9	13,8	0,045
for admitted patients	(9,4)	(16,2)	(10,2)	**(16,5)**	(10,4)	(13,1)	
Initial peritoneal cell count	5431	**2483**	**2650**	**3466**	**3240**	**2151**	0,0005
(per mm^3)	(6995)	**(2379)**	**(5276)**	**(5463)**	**(3004)**	**(3431)**	
Initial neutrophil count (%)	78,9	74,5	**72,1**	77,3	79,6	**68,8**	0,0005
	12,3	(12,7)	**(15,4)**	(14,0)	(14,9)	**(18,3)**	
Initial treatment (%)							0,0005
Ciprofloxacin	55,6	59,3	**75,5**	**69,5**	**44,9**	59,1	
Vanco + Cephalosporin	42,7	35,2	**21,1**	**26,5**	**53,5**	37,6	
Other/No data	1,7	5,5	**3,4**	**4,0**	**1,6**	3,3	
Final treatment (%)							0,0005
Ciprofloxacin	19,8	**16,6**	**36,1**	**33,7**	**3,3**	**40,3**	
Cephalosporin	28,3	**5,6**	**4,7**	**23,3**	**16,4**	**6,0**	
Carbapenem	1,4	**1,9**	**0**	**8,4**	**4,9**	**2,7**	
Vancomycin	33,8	**18,5**	**36,0**	**0**	**6,6**	**4,7**	
Antibiotic association*	15,0	**55,6**	**22,4**	**28,9**	**66,9**	**44,3**	
Other/No data	1,7	**1,8**	**0,8**	**5,7**	**1,9**	**2,0**	
Number of days of antibiotic	15,6	**22,1**	15,8	16,6	**19,7**	**12,9**	0,0005
therapy	(4,8)	**(8,0)**	(5,9)	(6,1)	**(5,2)**	**(5,1)**	

* Most common antibiotic associations Vancomycin + Cephalosporin/Rifampicin (Gram positive microorganisms) or Cephalosporin + Aminoglycoside (Gram negative microorganisms)

Figures denote % of cases (categorical variables) or mean (standard deviation)(numerical variables). Comparison by χ2 distribution and one-way ANOVA. P values denote overall significance. In bold, categories presenting a significant difference with streptococcal infections (Scheffé)

Keys: Strept: Streptococci; SAU: *Staphylococcus aureus*; CNS: Coagulase-negative staphylococci; GNB: Gram negative bacteria (*Enterobacteriaceae* + Nonfermenting Gram negative bacteria) ; Poly: Polymicrobial

Streptococcal PDrP associated the most severe initial inflammatory reaction of all the study groups, as estimated from the baseline peritoneal cell count and the proportion of polymorphonuclear leukocytes. Otherwise, the clinical presentation of streptococcal PDrP showed similarities to infections by CNSt and culture-negative PDrP. Final antimicrobial therapy of these infections was based on intraperitoneal ciprofloxacin (until 2007), cephalosporins and vancomycin; only 15,0% of cases were finally treated with an antibiotic association.

Clinical outcomes

The main clinical outcomes of PDrP are presented in Table 6. Remarkably, streptococcal PDrP carried the highest probability of uneventful continuation of PD. Catheter removal was less frequent than in any other type of PDrP, except culture-negative PDrP (p=0,04 versus CNSt). The risk of relapse, in opposition to the case of repeat infection (see above) was low, and treatment failure was less frequent than in any other group, although the difference with CNSt (p=0,072) and negative-culture PDrP (p=0,11) did not reach statistical significance.

Table 6

Clinical outcomes

	**Strept.**	**SAu**	**CNS**	**GNB**	**Poly**	**Culture-negative**	**P value**
Time to remission or catheter removal (days)	5,7 (3,9)	5,3 (3,6)	5,2 (3,5)	5,1 (4,7)	4,3 (2,2)	5,0 (2,9)	0,023
Catheter removed (%)	3,0	**31,6**	**7,0**	**23,1**	**8,1**	2,3	0,0005
Relapse (%)	10,7	8,8	**17,9**	**19,8**	9,5	7,6	0,003
Recurrence (%)							0,16
Other bacteria	0,4	0	2,1	4,4	0,7	1,8	
Yeasts	2,6	3,5	2,5	2,2	2,2	0,8	
Main outcomes (%)							0,0005
PD continued >3 months	97,0	**76,9**	96,1	**85,2**	91,9	94,0	
Drop-out to Hemodialysis	0	**8,8**	2,8	**6,6**	2,2	0,8	
PDrP-related death	3,0	**12,3**	1,1	**8,2**	5,9	5,3	
Treatment failure (%)	4,7	**42,1**	8,4	**26,9**	**11,1**	7,5	0,0005

Figures denote % of cases (categorical variables) or mean (standard deviation)(numerical variables). Comparison by χ2 distribution and one-way ANOVA. P values denote overall significance. In bold, categories presenting a significant difference with streptococcal infections (Scheffé)

Keys: Strept: Streptococci; SAU: *Staphylococcus aureus*; CNS: Coagulase-negative staphylococci; GNB: Gram negative bacteria (*Enterobacteriaceae* + Nonfermenting Gram negative bacteria); Poly: Polymicrobial

Table 7 presents the results of multivariate analysis for the main outcomes. Streptococcal PDrP carried a better prognosis than infections by SAu or GNB, and similar to CNSt and culture-negative infections.

Table 7

Predictors of clinical outcomes. Multivariate analysis

	OR	95% CI	P value
**CATHETER REMOVAL**			
Age (per year)	0,97	0,96-0,99	0,002
Time on PD at the time of infection (per month)	1,02	1,01-1,03	0,0005
PD vintage (Ref. 1990-99)			0,86
2000-2009	1,06	0,84-1,76	
2010-2019	0,78	0,36-1,43	
Causative agent of infection (Ref. *Streptococcus spp*)			
Staphylococcus aureus	10,85	4,09-18,82	0,0005
Coagulase-negative *Staphylococcus spp*	1,73	0,70-4,28	0,23
Gram negative bacteria	8,39	3,62-19,48	0,0005
Polymicrobial	2,19	0,81-5,92	0,12
Culture-negative	0,72	0,18-2,85	0,64
**PERITONEAL INFECTION-RELATED DEATH**			
Age (per year)	1,10	1,06-1,14	0,0005
Time on PD at the time of infection (per month)	1,03	1,02-1,04	0,0005
Charlson's score (per point)	1,15	1,03-1,33	0,019
Recent/Active immunosuppression	4,99	1,91-13,02	0,001
PD vintage (Ref. 1990-99)			0,80
2000-2009	1,53	0,70-3,36	
2010-2019	0,64	0,26-1,56	
Causative agent of infection (Ref. *Streptococcus spp*)			
Staphylococcus aureus	10,50	3,05-36,12	0,0005
Coagulase-negative *Staphylococcus spp*	0,25	0,06-1,02	0,054
Gram negative bacteria	2,74	1,03-7,30	0,041
Polymicrobial	1,66	0,55-5,03	0,36
Culture-negative	2,02	0,65-6,29	0,22
**TREATMENT FAILURE**			
Time on PD at the time of infection (per month)	1,02	1,01-1,03	0,0005
PD vintage (Ref. 1990-99)			0,45
2000-2009	1,22	0,78-1,92	
2010-2019	0,89	0,52-1,52	
Causative agent of infection (Ref. *Streptococcus spp*)			
Staphylococcus aureus	14,30	6,28-32,59	0,0005
Coagulase-negative *Staphylococcus spp*	1,41	0,66-3,00	0,38
Gram negative bacteria	6,90	3,41-13,90	0,0005
Polymicrobial	2,00	0,88-4,58	0,09
Culture-negative	1,76	0,98-2,31	0,22

Best models. Stepwise logistic regression analysis

Keys: OR: Odds ratio; CI: Confidence interval; PD: Peritoneal Dialysis; Treatment failure: At least one of: catheter removal, death or drop-out to hemodialysis for at least 3 months

Comparisons of PDrP by different species of streptococci

We compared the clinical presentation and outcomes of four different subgroups of streptococci, namely *mitis/oralis/sanguis/gordoni* (n=92), *salivarius/vestibularis* (n=85), other viridans (n=46) and non-viridans (n=12). We were unable to disclose any significant differences or trends, regarding the variables displayed in Tables 5 and 6 (data not presented).

Clinical significance of the isolation of streptococci in polymicrobial PI

As previously stated, at least one streptococcal strain was isolated in 78 of the 133 polymicrobial PDrP included in the analysis. In 28 cases, polymicrobial PDrP was caused by 2 different strains of streptococci. Polymicrobial PDrP with presence of streptococci presented with marginally higher peritoneal cell counts (3501 vs 2764 cells/mm^3, p=0,092) and % of neutrophils (81,9 vs 75,9%, p=0,031), than infections without participation of these bacteria. We did not detect any other clinical difference, between these subsets (data not presented).

Discussion

Our results provide clues to understand how the causative spectrum of PDrP may have evolved, during the last three decades. The systematic implementation of measures of prevention, essentially oriented to reduce touch contamination and catheter-related PDrP [8,22] resulted in a progressive decline of the incidence of infections by staphylococci and GNB (Table 3, Figure 1). On the contrary, in a setting of slowly decreasing incidence of PDrP, we observed clear trends to an increase in the rates of streptococcal and polymicrobial infections, with the particularity that a majority of the latter included isolation of streptococci. Overall, streptococci were isolated in 41,3% of the PDrP diagnosed between 2010 and 2019. Thinking in positive terms, this change may have been beneficial, because streptococcal PDrP associated better outcomes than infections by SAu or GNB (and similar to CNst) (Tables 6 and 7). From an opposite point of view, this evolution suggests that current prevention measures

may not be effective, to reduce the risk of streptococcal infections. This finding should not be completely unexpected. First, catheter-related streptococcal PDrP is an infrequent event (Table 5) [23,24], downplaying the capacity of catheter care to prevent this complication. Touch contamination is a potential source of streptococcal infection, but the ubiquity (skin, mouth, upper respiratory tract, upper and lower gastrointestinal tract) of this genus of bacteria [17] entails more potential foci of contamination and, probably, a higher capacity for hematogenous spread than CNSt. The circumstance that 35,8% of the patients who presented streptococcal PDrP suffered at least one other episode of infection by the same genus suggests a persistence and/or multiplicity of foci of infection. Other studies have detected a significant incidence of repeat streptococcal PDrP, although not to the extent observed in our study [14,15]. The oral cavity is a subject of particular concern as a source of streptococcal PDrP, due to a high degree of colonization by this family of bacteria [25] and to a potential for both hematogenous spread and direct contamination, particularly if face masks are used inappropriately during the PD exchange [8]. On the other hand, the high proportion of polymicrobial PDrP with isolation of streptococci [13,14,16] suggests that the lower gastrointestinal tract may be another significant source of infections by these bacteria. Unfortunately, we were unable to create a specific risk profile for streptococcal PDrP (Table 4), partly due to the nonavailability of potentially relevant variables, including bucodental care, adherence to the use of face masks during the PD changes or intestinal disorders. Older age, hypoalbuminemia and overweight associated a higher risk of streptococcal PDrP, but this predictive model lacked specificity, because these factors were also linked to the general risk of PDrP [26-28]. This inability to establish a specific predictive model for streptococcal PDrP was also observed in the best powered study so far [14].

Our results disclosed a higher relative incidence of streptococci in both monobacterial and polymicrobial PDrP than previous reports [11-14,16,29-32]. However, most of the cited studies investigated patients started on PD in the 1990s', when the incidence of streptococcal

PDrP was also lower in our centre. In addition, local factors, including differences concerning PD practices and the global incidence of PDrP, could help to explain this discrepancy. For instance, irregular adherence to measures with a demonstrated efficacy to prevent staphylococcal PDrP [33] may associate a lower incidence of streptococcal infections [14,34,35]. The latter factor could also explain why only some studies [12,13,36] were able to detect the trend to an increase in the incidence of streptococcal PDrP, so clearly disclosed by our analysis. The exclusion by protocol of rampant enteric PDrP and the high proportion of polymicrobial peritonitis with isolation of streptococci may help to explain why, in our study, the observed outcomes of these infections were more benign than usually reported (Table 6)[29,31].

The present study agrees, for the most part, with previous reports on the clinical presentation and outcome of streptococcal PDrP (Tables 5 and 6). These infections have a relatively severe initial presentation, with a marked inflammatory response, as shown by the baseline peritoneal cell counts (Table 5), and a relatively slow resolution (Table 6)[13]. However, initial aggressiveness is usually followed by complete recovery with antibiotic therapy alone, with low rates of hospital admission, catheter removal, relapse, recurrence and, most importantly, hard outcomes (mortality, technique failure)(Table 7)[13-16,18]. Previous studies have shown that the time course of peritoneal cellularity, rather than the initial count, is the best marker of outcome of PDrP [37,38]. Our data also confirm that these bacteria are usually susceptible to common antibiotic regimes [39], as either vancomycin, betalactams or fluoroquinolones were able to control streptococcal PDrP (Table 5)[14,15]. Current ISPD recommendations endorse the use of ampicillin, for this purpose [8] but, in our unit, the success of the antimicrobials used for initial management of PDrP explains why ampicillin was not a usual choice.

Our results do not suggest particularities for any subgroup of streptococci, regarding clinical presentation or outcomes. Isolation of *Streptococcus bovis* has been linked to an

increased risk of colorectal cancer. We recorded 5 instances of this infection, and all followed an uneventful clinical course, in agreement with a recent report [18]. We neither observed any significant diference among polymicrobial PDrP with or without isolation of streptococci. Previous studies have underlined that the isolation of GNB [31], intestinal anaerobics and *Enterococcus faecium* [40] may represent better markers of a complicated outcome of polymicrobial PDrP.

Our study suffers significant limitations, including a retrospective, single-centre design, which may overrate the effect of local factors. Some variables with a potential impact on the risk of streptococcal PDrP (e.g. oropharingeal disorders) could not be recorded. On the other side, significant strengths include the large time span, very appropriate to investigate time trends, and the high quality of our database, which permitted a complete analysis of the study population.

In summary, streptococci have progressively acquired a dominant role in the causative spectrum of PD-related PDrP. This circumstance has been favoured by simultaneous declines in the incidences of PD-related infections by staphylococci and GNB. This time course suggests that current measures for prevention of PDrP may not be sufficiently effective, for this genus of microorganisms. The facts that streptococcal PDrP associate a high risk of repeat infections and that streptococci are frequently isolated in polymicrobial infections reinforce this perception. Streptococcal PDrP are characterized by an initially aggressive clinical course and a relatively slow resolution, but also by a usually uneventful outcome with antibiotics alone. Future research will be necessary to improve prevention of infections by this genus of Gram positive bacteria.

Acknowledgments: None to report

References

1. Perl J, Douglas S, Bieber BA, Boudville N, Kanjanabuch T, Ito Y: Peritoneal Dialysis-Related Infection Rates and Outcomes: Results From the Peritoneal Dialysis Outcomes and Practice Patterns Study (PDOPPS). Am J Kidney Dis 2020; doi: 10.1053/j.ajkd.2019.09.016
2. Ye H, Zhou Q, Fan L, Guo Q, Mao H, Huang F et al: The impact of peritoneal dialysis-related peritonitis on mortality in peritoneal dialysis patients. BMC Nephrol 2017; 18: 186
3. Kolesnyk I, Dekker FW, Boeschoten EW, Krediet RT: Time dependent reasons for peritoneal dialysis technique failure and mortality. Perit Dial Int 2010; 30: 170-177
4. Chidambaram M, Bargman JM, Quinn RR, Austin PC, Hux JE, Laupacis A: Patient and physician predictors of peritoneal dialysis technique failure: a population based, retrospective cohort study. Perit Dial Int 2011; 31: 565-573
5. Kumar VA, Sidell MA, Yang WT, Jones JP: Predictors of peritonitis, hospital days, and technique survival for peritoneal dialysis patients in a managed care setting. Perit Dial Int 2014; 34: 171-178
6. Htay H, Cho Y, Pascoe EM, Darssan D, Nadeau-Frechette AC, Hawley C et al: Center Effects and Peritoneal Dialysis Peritonitis Outcomes: Analysis of a National Registry. Am J Kidney Dis, 2018; 71: 814-821
7. Pulliam J, Li NC, Maddux F, Hakim R, Finkelstein FO, Lacson E: First year outcomes of incident peritoneal dialysis patients in the United States. Am J Kidney Dis 2014; 64: 761-769
8. Li PKT, Szeto CC, Piraino B, Arteaga J, Fan S, Figueiredo AE et al: ISPD Peritonitis Recommendations: 2016 Update on Prevention and Treatment. Perit Dial Int, 2016; 36: 481-508
9. Htay H, Johnson DW, Wiggins KJ, Badve SV, Craig JC, Strippoli GF et al: Biocompatible dialysis fluids for peritoneal dialysis. Cochrane Database Syst Rev, 2018. 10(CD007554).

10. Nataatmadja M, Cho Y, Johnson DW: Continuous Quality Improvement Initiatives to Sustainably Reduce Peritoneal Dialysis-Related Infections in Australia and New Zealand. Perit Dial Int 2016; 36: 472-477
11. van Esch S, Krediet RT, Struijk DG: 32 years' experience of peritoneal dialysis-related peritonitis in a university hospital. Perit Dial Int 2014; 34: 162-170
12. Zelenitsky SA, Howarth J, Lagacé-Wiens P, Sathianathan C, Ariano R, Davis C et al: Microbiological Trends and Antimicrobial Resistance in Peritoneal Dialysis-Related Peritonitis, 2005 to 2014. Perit Dial Int 2017; 37: 170-176
13. Shukla A, Abreu Z, Bargman JM: Streptococcal PD peritonitis--a 10-year review of one centre's experience. Nephrol Dial Transplant 2006; 21: 3545-3549
14. O'Shea S, Hawley CM, McDonald SP, Brown FG, Rosman JB, Wiggins KJ: Streptococcal peritonitis in Australian peritoneal dialysis patients: predictors, treatment and outcomes in 287 cases. BMC Nephrol 2009; 10: 19
15. Chao CT, Lee SZ, Yang WS, Chen HW, Fang CC, Yen CJ: Viridans streptococci in peritoneal dialysis peritonitis: clinical courses and long-term outcomes. Perit Dial Int 2015; 35: 333-341
16. Liu Y, Chen BC, Liu JW, Chen CJ, Kuo LC, Chang WX et al: Viridans Streptococcus peritonitis in peritoneal dialysis: Clinical characteristics and comparison with concurrent polymicrobial infection. BMC Nephrol 2018; 19: 271
17. Ruoff KL, Bisno AL: Classification of streptococci. In: Mandell, Douglas & Bennett's Principles and Practice of Infectious Diseases. JE Bennett, R Dolin and MJ Blaser (eds.). Saunders, Philadelphia 2015; 2283-2284
18. Yap DYH, To KKW, Yip TPS, Lui SL, Chan TM, Lai KN et al: Streptococcus bovis complication peritoneal dialysis. A review of 10 years' experience. Perit Dial Int 2012; 32: 55-59

19. Pérez-Fontán M, García Falcón T, Rosales M, Carmona AR, Adeva M, Lozano IR et al: Treatment of Staphylococcus aureus nasal carriers in continuous ambulatory peritoneal dialysis with mupirocin: long-term results. Am J Kidney Dis 1993; 22: 708-712
20. Piraino B, Bailie GR, Bernardini J, Boeschoten E, Gpta A, Holmes C et al: Peritoneal dialysis-related infections recommendations: 2005 update. Perit Dial Int 2005; 25: 107-131
21. Kim HJ, Fay MP, Feuer EJ, Midthune DN: Permutation tests for jointpoint regression with applications to cancer rates. Stat Med 2000; 19: 335-351 (correction: 2001; 20: 655)
22. Szeto CC, Li PKT, Johnson DW, Bernardini J, Dong J, Figueiredo AE et al: ISPD catheter-related infection recommendations: 2017 Update. Perit Dial Int 2017; 37: 141-154
23. Zhang L, Badve SV, Pascoe EM, Beler E, Cass A, Clark C et al: The Effect of Exit-Site Antibacterial Honey Versus Nasal Mupirocin Prophylaxis on the Microbiology and Outcomes of Peritoneal Dialysis-Associated Peritonitis and Exit-Site Infections: A Sub-Study of the Honeypot Trial. Perit Dial Int 2015; 35: 712-721
24. Santos C, Pérez-Fontán M, Rodríguez-Carmona A, Calvo M, López Muñiz A, López-Calviño B et al: Identification of Targets for Prevention of Peritoneal Catheter Tunnel and Exit-Site Infections in Low Incidence Settings. Perit Dial Int 2016; 36: 43-51
25. Abranches J, Zeng I, Kajfasz JK, Palmer SR, Chakraborty B, Wen ZT et al: Biology of Oral Streptococci. Microbiol Spectr 2018; doi: 10.1128/microbiolspec.GPP3-0042-2018
26. Wang Q, Bernardini J, Piraino B, Fried L: Albumin at the start of peritoneal dialysis predicts the development of peritonitis. Am J Kidney Dis 2003; 41: 664-669
27. McDonald SP, Hawley CM, Rumpsfeld M, Johnson DW: Obesity is a risk factor for peritonitis in the Australian and New Zealand peritoneal dialysis patient populations. Perit Dial Int 2004. 24: 340-346
28. Pérez Fontan M, Rodriguez-Carmona A, García-Naveiro R, Rosales M, Villaverde P, Valdés F: Peritonitis-related mortality in patients undergoing chronic peritoneal dialysis. Perit Dial Int 2005; 25: 274-284

29. Kim GC, Korbet SM: Polymicrobial peritonitis in continuous ambulatory peritoneal dialysis patients. Am J Kidney Dis 2000; 36: 1000-1008
30. Mujais S: Microbiology and outcomes of peritonitis in North America. Kidney Int (Suppl) 2006; 103: S55-S62
31. Barraclough K, Hawley CM, McDonald SP, Brown FG, Rosman JB, Wiggins KJ et al: Polymicrobial peritonitis in peritoneal dialysis patients in Australia: predictors, treatment, and outcomes. Am J Kidney Dis 2010; 55: 121-131
32. Ghali JR, Barraclough K, Brown FG, Rosman JB, Wiggins KJ, Johnson DW et al: Microbiology and outcomes of peritonitis in Australian peritoneal dialysis patients. Perit Dial Int, 2011; 31: p. 651-662
33. Boudville N, Johnson DW, Zhao J, Bieber BA, Pisoni RL, Piraino B et al: Regional variation in the treatment and prevention of peritoneal dialysis-related infections in the Peritoneal Dialysis Outcomes and Practice Patterns Study. Nephrol, Dial & Transplant 2019; 34: 2118-2126
34. Govindarajulu S, Hawley CM, McDonald SP, Brown FG, Rosman JB, Wiggins KJ et al: Staphylococcus Aureus Peritonitis in Australian Peritoneal Dialysis Patients: Predictors, Treatment, and Outcomes in 503 Cases. Perit Dial Int 2010; 30: 311-319
35. Burke M, Hawley CM, Badve SV, McDonald SP, Brown FG, Boudville N: Relapsing and recurrent peritoneal dialysis-associated peritonitis: a multicenter registry study. Am J Kidney Dis 2011; 58: 429-436
36. Rocha A, Rodrigues A, Teixeira L, Carvalho MJ, Mendonça D, Cabrita A: Temporal Trends in Peritonitis Rates, Microbiology and Outcomes: The Major Clinical Complication of Peritoneal Dialysis. Blood Purif 2012; 33: 284-291
37. Chow KM, Szeto CC, Cheung KK, Leung CB, Wong SS, Law MC: Predictive value of dialysate cell counts in peritonitis complicating peritoneal dialysis. Clin J Am Soc Nephrol; 2006; 1: 768-773

38. Xu R, Chen Y, Luo S, Xu Y, Zheng B, Zheng Y: Clinical characteristics and outcomes of peritoneal dialysis-related peritonitis with different trends of change in effluent white cell count: a longitudinal study. Perit Dial Int 2013; 33: 436-444

39. Haenni M, Lupo A, Madec JY: Antimicrobial resistance in Streptococcus spp. Microbiol Spectr 2018; Mar;6(2). doi: 10.1128/microbiolspec.ARBA-0008-2017

40. Ribera-Sánchez R, Perez Fontan M, López-Iglesias A, García-Enríquez A, Rodríguez-Carmona A: Comprehensive Approach to Peritoneal Dialysis-Related Peritonitis by Enteric Microorganisms. Comparison Between Single Organism and Polymicrobial Infections. Perit Dial Int 2018; 38: 139-146

Figure 1. Relative incidence of peritoneal infection by different causative microorganisms during six consecutive 5-year periods (upper), and average annual percent changes (AAPC) in the absolute rates of incidence during the 30-year study period.

Conflict of interest statement: None to be reported

Figure 1

Click here to access/download;Figure;aaaaFigure1_POne.jpg

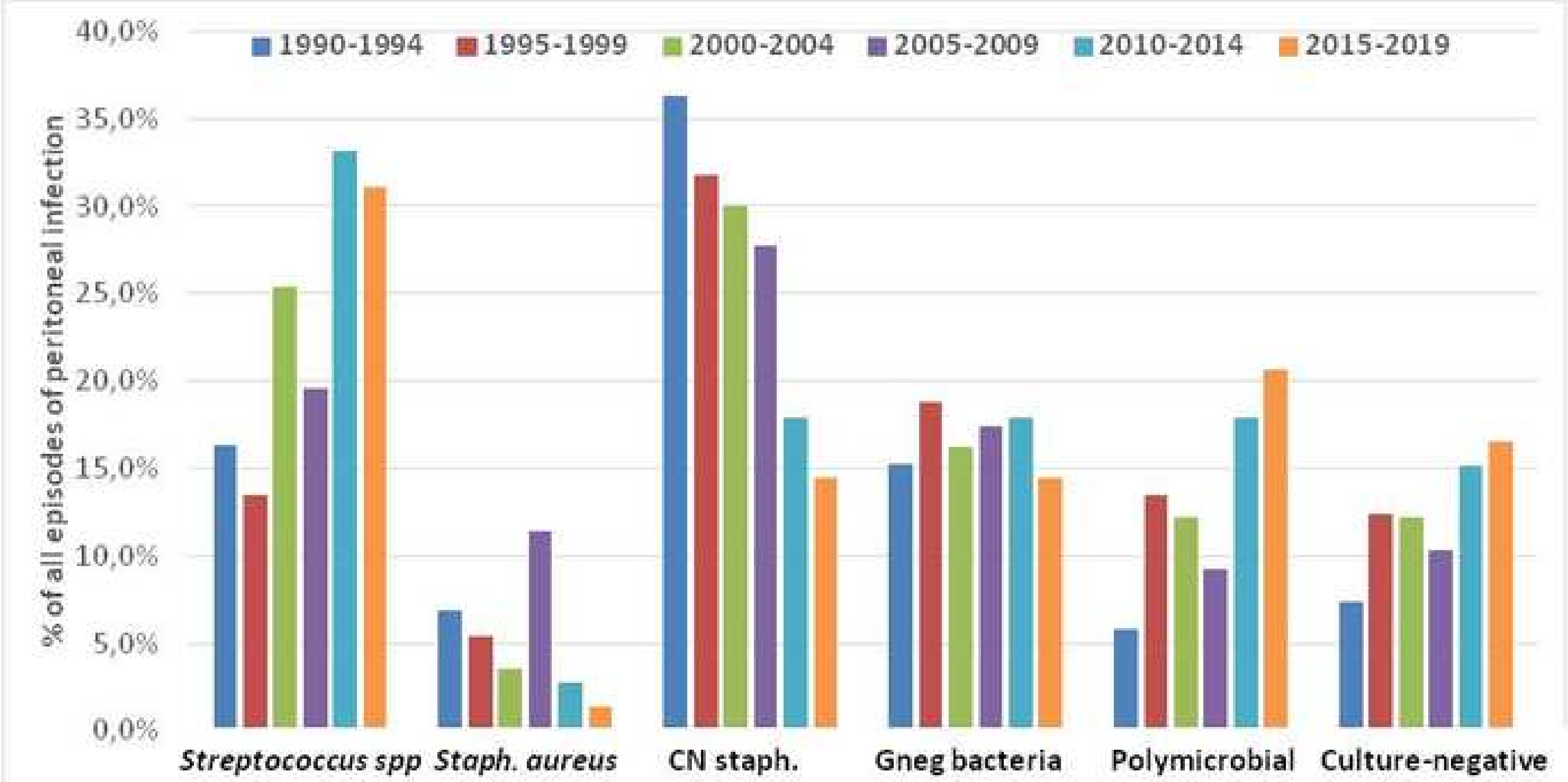

	AAPC	95% CI	p for trend
All episodes	-2,0	-3,2/-0,8	<0,05
Streptococcus spp	+1,6	-0,1/+3,2	NS
Staphylococcus aureus	-9,0	-15,7/-1,7	<0,05
Coagulase-negative staphylococci	-4,5	-6,2/-2,7	<0,05
Gramnegative bacteria	-2,0	-3,4/-0,6	<0,05
Polymicrobial	+1,8	+0,1/+3,5	<0,05
Culture-negative	+0,5	-1,5/+2,6	NS
