# Supplementary material for: Long-term trends in the incidence of peritoneal dialysis-related peritonitis disclose an increasing relevance of streptococcal infections: A longitudinal study
Source: PLoS One. 2020 Dec 21;15(12):e0244283. doi: 10.1371/journal.pone.0244283 (PMC7751873; doi:10.1371/journal.pone.0244283)
Supplement: Attachment — Submitted filename: Response_to_reviewers.docx [file pone.0244283.s002.docx]

Thank you for considering our study on “The Growing Role of Streptococci as Causative Agents of Peritoneal Dialysis-related Peritoneal Infections. A Longitudinal Analysis” (PONE-D-20-22429). We are now submitting revised version of the manuscript, in which we have done our best to answer the concerns and follow the recommendations of the reviewers. According to your instructions, we include a marked-up copy of the paper with the modifications introduced (red for deletions, blue for additions), as also a clean copy. We shall now state in detail the modifications introduced and the answers to the reviewers.

1) Editorial Board

*Please include the following items when submitting your revised manuscript:*

- *A rebuttal letter that responds to each point raised by the academic editor and reviewer(s). You should upload this letter as a separate file labeled 'Response to Reviewers'.*
- *A marked-up copy of your manuscript that highlights changes made to the original version. You should upload this as a separate file labeled 'Revised Manuscript with Track Changes'.*
- *An unmarked version of your revised paper without tracked changes. You should upload this as a separate file labeled 'Manuscript'.*
- **We have adapted the format of the manuscript to the requirements of the Journal. Figure 1 has been changed to a TIF format, as requested.**
- **We now state in the paper that oral informed consent was obtained only for the minority of patients who were accessible at the time of the study. In these cases, consent was approved by the Ethical Committee, and registered in the clinical records of the patients. For nonavailable patients, permission was given by the ethical Committee, under the condition of full de-identification of the cases.**
- **Regarding the question of open access to the data sets, sorry, this is not possible under Spanish or Galician regional data protection acts, even if the data bases are de-identified. However, this information can be obtained on request, needing only a previous approval by the local ethical committee. This information should be requested through the Galician Health System (Servicio Galego de Saúde, Sergas), in this address:**

**Dr. Celia Cal Purriños**

**Secretary of the Research Ethics Committee of the A Coruña-Ferrol Area**

**Rede Galega de Comités**

**Xerencia do Servizo Galego de Saúde**

**Edificio Administrativo San Lázaro s/n**

**15703 SANTIAGO DE COMPOSTELA (Spain)**

**Mail:** [**ceic@sergas.es**](mailto:ceic@sergas.es)

**Please, remember that the code number of the study for the Ethical Committee is 2020/190.**

2) Reviewer #1

**We warmly thank the kind general comments of the reviewer. Regarding the concerns and suggestions:**

- *Overview
  The authors have conducted a 30-year retrospective chart review to evaluate trends in incidence of PD-related peritonitis. They have found decreasing trends in staphylococci and Gram negative bacterial infections and an increase in streptococcal and polymicrobial infection. There wasn’t a difference in predictive risk factors for Streptococcal PDrP but had more favorable outcomes. There were no differences amongst species of Streptococci. This is information that is useful to clinicians, infection prevention and improving quality of care. The authors have described the various aspects of this study succinctly and performed an excellent review of why this is an issue of practical importance. They also highlight, the potential difficulties with interpreting this data due to the retrospective nature of the work many years later.

  Importance
  The submission is an important contribution as it highlights important etiological trends in PDrP and advocates for further research towards preventing streptococcal and polymicrobial infections.

  Abstract
  Appropriate wording and description.
  Throughout the manuscript, period has been replaced by a comma probably related to a software issue – example please change 1,6% to 1.6%*

**We have replaced commas by periods to express decimals, as requested**

- *Please reword “We disclosed significant trends to a decrease” and “The last three decades have contemplated a growing involvement”.
  Throughout the manuscript, please replace “associated a” with “was associated with a”*
  **Thank you for the style corrections in the Abstract and text. We have implemented them, as suggested**
- *Introduction
  Written well. No significant changes recommended.
  Please reword the last paragraph as it is wordy and unclear “Our main interest was to pursue a clinical perception that advances in the prevention and management of PDrP could have modified the clinical scenery of PD-related peritonitis, conferring streptococci a progressively dominant role, with potential consequences for the clinical presentation, management and outcomes of these infections.”*
  **We have reworded the last paragraph of the Introduction, as requested. We fully agree that the original one was wordy and confusing**
- *Methods
  The general design, study variables and outcomes are appropriate.
  Please delete “(primary objectives too)”*
  **In Method, we have deleted “primary objectives too”, as requested**
- *Is information regarding methicillin resistance available for Staphylococcus aureus? If so, please include it. If not available, please note that it is not available.*
  **In Method-Study Variables and Strategies, second paragraph, we have included a sentence indicating that 4 episodes of *Staph. aureus* infection were caused by methicillin-resistant strains. This small group was not a subject of individualized analysis, as now stated.**
- *Describe outpatient diagnosis, and management of PDrP and if any changes have been made over the last 30 years. Since hospital admission for PDrP is indicated for high-risk patients, infections with an aggressive clinical presentation, treatment failure after oriented antimicrobial therapy and isolation of microorganisms demanding in centre management – what is the management for those that don’t meet these criteria? Are cultures obtained prior to empiric therapy as an outpatient? This may have a significant impact on epidemiology, etiology, presentation and outcomes.*
  **In Method-Study Variables and Strategies, last two paragraphs, we have made changes to explain better the diagnostic procedures and outpatient management criteria, as requested**
- *Are chlorhexidine baths/ wipes used for any patients?*
  **Sorry, our procedures do not include clorhexidine baths/wipes**
- *Please cite SPSS per guideline*
  **We now cite the software as IBM-SPSS**
- *Results
  Four hundred and sixty-two patients (52.6%) suffered at least one episode of PDrP. This appears to be inconsistent.
  To clarify, did 462 out of 878 patients evaluated have a total of 1061 episodes of PDrP? If so, could this be stated more clearly for the reader?*
  **We have changed the text at the start of Results, to clarify the percentages, as suggested**
- *Antibiotic association should be delineated better. Is the implication deviation from standard practice?*
  **Regarding antibiotic associations, the main ones are depicted as a footnote in Table 5. We were not more specific because there were many different combinations. These associations were within common standards, but we used different betalactamics, amynoglycosides, glycopeptides and so on, and there were changes in the course of treatment in some cases. In our opinion, describing all this in detail does not have a major interest, and could result in a really wordy paragraph. We have decided not to change this, but will reconsider, if you request us to do so.**
- *Discussion
  Well described. No significant changes recommended.
  Given the suspicion for enteric source due to Streptococcal, polymicrobial and Gram negative infections even after exclusion of an obvious surgical etiology, it may help to include how many of these instances had negative abdominal imaging (if information available).*
  **Sorry, information on how many patients had a negative (normal) abdominal imaging is not available, although we could say “a majority”. This is an interesting question. We routinely perform abdominal imaging studies in all cases of polymicrobial peritoneal infection with involvement of anaerobics or *Enterobacteriaceae*, as also in monobacterial infections by *Enterobacteriaceae* or *Staph. aureus* following a torpid or aggressive clinical course. In our experience, both ultrasonography and CT scan offer a relatively low sensitivity to detect surgical conditions. However, we continue to perform these studies, because they are very orientative, when positive.**
- *With streptococcal PDrP, could presentation be delayed due to a sub-acute onset of illness and therefore present with a higher PD fluid cell count?*
  **Regarding clinical presentation of streptococcal PDrP, our experience, clearly reflected in the manuscript, suggests that these infections have an explosive, rather than subacute onset. The fact that they appear (not so much) later than other infections may reflect the earlier appearance of staphylococcal infections, which are often a consequence of a poor technique performance.**
- *Acknowledge limitation that therapy used may be broader than necessary for streptococcal PDrP. Since this paper evaluates outcomes, this may be important.*
  **We have included a new sentence in the Limitations paragraph of the Discussion, to recognize that therapy used for streptococcal infections may have been broader than necessary, as requested.**
- *Table 1-7; Figure 1
  Clear and appropriately defined. For figure, can consider trend line chart.*

**As refers to Figure 1, we have tested the lines and the bars options, and the latter looked clearly better**

3) Reviewer #2

We also thank the general comments of this reviewer. Regarding the specific comments:

- *The authors seem to fall between two stools in their methodology. The stated aim is particular focus on streptococcal infections, but they present data on all common forms of peritonitis, so it is only in the discussion that any real differences in approach appear. Consider altering the focus to a general overview of the peritonitis experience (the most interesting choice for this reviewer). I am not clear why they have chosen streptococci as the primary subject of investigation. If they wish to maintain the focus on streptococci, perhaps just comparing streptococci with “all others”.*
  **The reason why we had a particular focus on streptococcal infections was a perception, supported by preliminary data, that the relevance of these microorganisms as causative agents of PDrP had increased progressively during the last decades. However, we agree with the reviewer that this was not just a question of increasing streptococcal infections (in fact, this was close, but did not reach statistical significance), but also of a reciprocal decline in the incidence of staphylococcal and Gram negative infections. This is why a general analysis was necessary, to present adequately the question. We have modified the last paragraph of the Introduction, to be more inclusive.**
- *The incidence of streptococcal infections is more or less unchanged, varying between 0,063 and 0,122/year for a relatively small number of cases. This is confirmed by the joinpoint analysis. The title is thus somewhat misleading, and could really be called “The falling incidence of staphylococci…..”. The relative incidence of streptococci and staphylococci is uninteresting in this respect.*
  **The incidence of streptococcal infections showed a clear trend (close to significance by joinpoint regression) to an increase. You should also consider that polymicrobial infections (with isolation of streptococci in 58.6% of the cases) also increased during the study period. The remarkable point is that, while staphylococcal and Gram negative infections decreased (both in absolute and relative terms), streptococcal infections (and their polymicrobial “associates”) tended to increase, significantly (relative) or close to significance (absolute). This facts demand explanation and, likely, changes in procedures.**
- *Several of the variables do not seem to be normally distributed, e.g. Charlson Comorbidity score, peritoneal cell count, time to remission or catheter removal, but are presented and analysed as such in the multivariate analyses.*
  **Regarding the distribution of some variables, this question was decided by our epidemiologist (SPD). It is true that some variables (e.g. Charlson, GFR or peritoneal cell count) did exhibit some deviation from normal distribution. However, the deviation was not marked, and the sample was large enough to permit analyses by parametric tests. The only exception was C reactive protein, which exhibited a** **markedly abnormal distribution (Table 1), and had to be categorized for analysis.**
- *Some of the statistics “feel” wrong. E.g an unchanged Strept. peritonitis incidence translates into a 77% increase in Table 4. A more or less identical time to remission is significant in Table 6. Please check for common statistical errors (maybe using professional statistical assistance), e.g. failing to correct for multiple comparisons, using parametric analyses for non-parametric variables, treating categorical variables as continuous variables.*
  **We have checked the potential errors in statistic analysis, and believe that the presented figures are correct. In the case of *Streptococcus* incidence, the change was not significant, but close to, in univariate analysis. Table 4 presents adjusted results (multivariate), and comparisons are made against the period 1990-99 (lowest incidence), while joinpoint regression analyzes the whole period. On the other side, significance in line 1 of Table 6 (time to remission) represents the result of the omnibus ANOVA test for all the categories (by the way, p value was 0.033 and not 0.023, sorry). Please, note that there is no figure in bold, indicating that, in post hoc analyses, streptococci were not the source of this significant difference.**
- *Page 8 ” including 25 patients (15,8%) with more than two monobacterial streptococcal PDrP during follow-up (range 3-7).” Slightly unclear. 3-7 months? What about patients with two monobacterial streptococcal PDrP during follow-up?*
  **In page 8, we have clarified the figures for repeat streptococcal infections, according to the request of the reviewer. We hope this is adequately clarified**
- *Page 9. “Median delays to the first episode of PDrP were 8 (interquartile range 3 to 17) (overall) and 12 months (interquartile range 5 to 24)(streptococcal PDrP).” Does “overall” include or exclude streptococcal PDrP?*

**In the last sentence of the previous paragraph (median delays to the first episode of PDrP), overall includes streptococcal infections**

- *Page 9 “On the other hand, at least one streptococcal strain was isolated in 78 polymicrobial infections”. Table 2 seems to state 116 patients. Unclear, please clarify.*

**In page 9, the disagreement is ony apparent: 116 different strains of streptococci were isolated during 78 episodes of polymicrobial peritonitis. It was not uncommon to isolate 2 or 3 (even 4, in one case) different streptococcal strains in the same episode**

- *“No patient denied consent”. In this long-term retrospective study, requirement for patient consent was presumably waived?*

**The reviewer is fully right about the question of informed consent. Only a minority of patients were accessible at the time of the study. For the others, we obtained a permission of the Ethical Committee. We have made a change in the last paragraph of Method-General Design, to clarify this.**

- *Table 4. Four outcomes are defined in the Methods, but only three are presented. Were the outcomes censored for each other?*

**The four outcomes mentioned are depicted at the bottom of Table 6**

Again, we thank the reviewers for their attention, their kind comments and, most importantly, for their interesting and consequential concerns and recommendations. We hope that the revised manuscript is satisfactory, but will readily consider any new suggestion or recommendation.

Sincerely

Miguel Pérez Fontán
