# Supplementary material for: Long-term trends in the incidence of peritoneal dialysis-related peritonitis disclose an increasing relevance of streptococcal infections: A longitudinal study
Source: PLoS One. 2020 Dec 21;15(12):e0244283. doi: 10.1371/journal.pone.0244283 (PMC7751873; doi:10.1371/journal.pone.0244283)
Supplement: Attachment — Submitted filename: aaaaaResponse_to_reviewers_R2.docx [file pone.0244283.s003.docx]

Thank you for considering the revised version of our study on “The Growing Role of Streptococci as Causative Agents of Peritoneal Dialysis-related Peritoneal Infections. A Longitudinal Analysis” (PONE-D-20-22429). We are now submitting a secondly revised version of the manuscript, addressed to answer the remaining concerns. According to the instructions, we include a marked-up copy of the paper with the modifications introduced (red for deletions, blue for additions), as also a clean copy. We shall now state in detail the modifications introduced and the answers to the reviewers.

1) Editorial Board

We understand that there is no new specific concern or query.

2) Reviewer #1

As in the previous case, we understand that there is no specific concern or query. We thank the reviewer for his/her contributions.

3) Reviewer #2

We also thank the relevant concerns manifested by this reviewer. First, we want to state that, to our belief, the combination of clear trends to an increase in the incidence of streptococcal monobacterial (even if not significant!), and polymicrobial infections (significant and with a prominent involvement of streptococci) permits to speculate that streptococcal infections may have partly occupied the niche left by staphylococci and GNB. In any case, we also feel that the changes proposed by the reviewer are very reasonable. These changes downplay, but do not distort the original conclusions of the paper, and we have decided to follow the recommendation. Consequently, we have modified the title of the manuscript, the Conclusions of the Abstract, the Discussion (just one word deleted to downplay the contention) and the Summary, at the end of the Discussion. We hope that these new changes to the presentation are satisfactory.

Again, we thank the reviewers for their attention, their kind comments and, most importantly, for their interesting and consequential concerns and recommendations. We hope that the secondly revised manuscript is now satisfactory, but will readily consider any new suggestion or recommendation.

Sincerely

Miguel Pérez Fontán
